# Niches of three sympatric montane ground‐dwelling squirrels: Relative importance of climate, topography, and landcover

**DOI:** 10.1002/ece3.9949

**Published:** 2023-03-31

**Authors:** Aviva J. Rossi, Robert C. Klinger, Elise C. Hellwig, Dirk H. Van Vuren

**Affiliations:** ^1^ Department of Wildlife, Fish, & Conservation Biology University of California, Davis One Shields Avenue Davis California 95616 USA; ^2^ Western Ecological Research Center U.S. Geological Survey 2761 Glenbrook Way Bishop California 93514 USA

**Keywords:** *Callospermophilus lateralis*, ecological factor analysis, ENFA, *Marmota flaviventer*, niche, Sierra Nevada, squirrel, *Urocitellus beldingi*

## Abstract

Species with different ecological niches will likely exhibit distinct responses to a changing environment. Differences in the magnitude of niche specialization may also indicate which species may be more vulnerable to environmental change, as many life‐history characteristics are known to affect climate change vulnerability. We characterized the niche space of three sympatric high‐elevation ground‐dwelling squirrels, yellow‐bellied marmot (*Marmota flaviventer*), Belding's ground squirrel (*Urocitellus beldingi*), and golden‐mantled ground squirrel (*Callospermophilus lateralis*), in the alpine and upper subalpine regions of the Sierra Nevada in California. We used 5879 observations of individual squirrels, collected from 4 years (2009–2012) of transect survey data, to quantify which ecogeographical variable types (climate, topography, or landcover) were most important in defining the niche of each species. We conducted Ecological Niche Factor Analysis to quantify the niche and generate indices of “marginality” (magnitude of selection) and “specialization” (narrowness of niche space). All three species demonstrated differential use of niche space when compared to the available niche space. Moreover, the relative importance of the variables shaping the niche differed among these species. For example, the presence of meadows was important in defining the niche for *U. beldingi* and *M. flaviventer*, but the presence of conifers was important to *C. lateralis*. Precipitation was important in defining the niche for all three species, positively so for *U. beldingi*, and negatively for the other two species. The niche breadth of these three species was also positively associated with geographic range size. Mammals in high‐elevation mountain systems often are perceived as vulnerable to climate shifts, but our results underscore the importance of also including non‐climate‐based factors in defining the niche. The overall magnitude of niche selection for all three species was driven by a combination of topographic, climatic, and landcover factors; thus, efforts to forecast areas where these species can persist in the future need to evaluate from more than just a climatic perspective.

## Graphical abstract

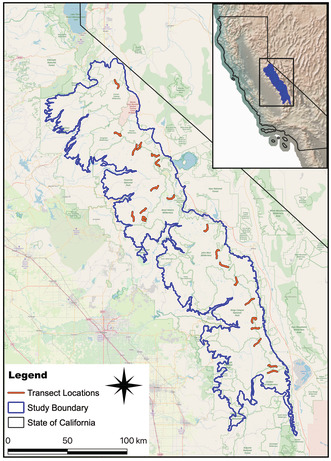

We characterized the niche space of three sympatric high‐elevation ground‐dwelling squirrels, yellow‐bellied marmot (*Marmota flaviventer*), Belding's ground squirrel (*Urocitellus beldingi*), and golden‐mantled ground squirrel (*Callospermophilus lateralis*), in the alpine and upper subalpine region of the Sierra Nevada in California. All three species demonstrated differential use of niche space and the relative importance of the variables shaping the niche differed among these species. The niche breadth of these three species was also positively associated with geographic range size.

## 1 INTRODUCTION

Understanding the distribution of species is one of the fundamental goals of ecology (Andrewartha & Birch, 1986; Smith et al., 2008). At broad spatial extents, environmental conditions, including climate and habitat, determine where a species can persist (Lomolino et al., 2006; Peterson et al., 2011). Indeed, the spatial distribution of environmental conditions, currently and historically, determines the potential geographic distribution of a species (Peterson et al., 2011). Ecological niche theory has been used to describe and quantify the range of environmental conditions where a species is likely to persist (Chase & Leibold, 2003; Peterson et al., 2011). Of particular importance are the Grinnellian niche, which emphasizes the interplay between behavior, environmental conditions, and the geographic distribution of a species (Grinnell, 1917), and the Hutchinsonian niche (Hutchinson, 1944), in which variables related to resources and environmental conditions comprise vectors that define an n‐dimensional hyper‐volume representing the niche space. Although the conceptual foundations of the Grinnellian and Hutchinsonian niches differ, they provide complementary theoretical frameworks for describing and measuring niche space and species distributions, especially regarding changes in range boundaries (Peterson et al., 2011).

Quantifying niche space for species in a given community can help forecast how the community might change under altered environmental conditions, as is expected in an era of global climate change. Anthropogenic climate change is affecting most ecosystems worldwide (IPCC, 2014). Species can be affected directly by increased temperature (physiological restrictions) and indirectly by factors such as altered snow and ice cover, seasonal availability of water, precipitation, and changes to vegetation distribution (IPCC, 2014; Morelli et al., 2011). Species can show notable niche conservation over geologic time (Peterson et al., 1999), and are therefore unlikely to respond to current environmental changes by changing their life‐history requirements (Bennett, 1997; but see Davis et al., 2005; Smith et al., 2019). Species have responded in several ways to past climate change, including changes in phenology (Socolar et al., 2017) and range shifts into areas with suitable conditions (Bennett, 1997; Davis & Shaw, 2001; Graham, 1986; Graham et al., 1996; Inouye et al., 2000; Parmesan, 2006).

Climate change effects are expected to be especially pronounced on high‐elevation species because of their specialized physiology and geographical constraints to their range (Erb et al., 2011; La Sorte & Jetz, 2010; Parmesan, 2006). The Sierra Nevada mountain range in the western United States, with 12 peaks >4000 m in elevation, has experienced shifts in temperature, precipitation, snowpack, and hydrology over the past several decades (Cayan et al., 2001; Dettinger et al., 2018; Dettinger & Cayan, 1995; Mote et al., 2005; Thorne et al., 2007). Changes in the distributions of some mammals in this region have already been noted (Rowe et al., 2015) and further changes to resident species have been predicted in the near future (Dettinger et al., 2018). However, environmental conditions besides climate, such as topography and landcover type also influence species distributions. These conditions may either change at different rates than climate (e.g., landcover) or remain largely unchanged (e.g., topography). This suggests that species whose current distributions are driven primarily by climate would be vulnerable to changes in temperature and precipitation, but species whose distributions are shaped by a more complex interplay among climate, topography, and landcover would be less vulnerable.

High‐elevation mammals that depend on meadows face additional risks due to climate change. Snowfields in the Sierra Nevada have been diminishing in size, extent, and seasonal duration (Stewart et al., 2005). Projected reductions in snowpack in the Sierra Nevada will result in up to 54% reductions in winter snowmelt, relative to the late 1900s (Kim et al., 2009), leading to a drier hydrologic regime. This, in turn, could result in the reduction or loss of many high‐elevation meadows, or their colonization and eventual domination by shrubs and trees (Dullinger et al., 2003; Jakubos & Romme, 1993; Norman & Taylor, 2005).

Understanding the niche space for high‐elevation species is fundamental to understanding the type of environmental changes different species may be most affected by and thus which may be most vulnerable to climatic changes. Our goal in this study was to quantify and compare the Hutchinsonian niches of three sympatric species of ground‐dwelling squirrels that occur in the high‐elevation regions of the Sierra Nevada range—Belding's ground squirrel (*Urocitellus beldingi*), yellow‐bellied marmot (*Marmota flaviventer*), and golden‐mantled ground squirrel (*Callospermophilus lateralis*). These species of ground‐dwelling squirrels have previously been modeled to be at high to very high risk due to climate change (McCain, 2019). Quantitative studies of the basic biology (excluding physiology and behavior, which are comparatively well‐studied (e.g., Wells et al., 2022)) of these species of high‐elevation ground squirrels specific to the Sierra Nevada range are surprisingly limited (Bronson, 1979; Sherman & Morton, 1984); and relatively few studies have provided the basic life‐history information needed to make predictions in the face of climate change or addressing climate change effects (Moritz et al., 2008; but see Morelli et al., 2012). These three species are seasonal hibernators that have some overlap in habitat use and diet; however, most of the information regarding the habitat requirements is qualitative or limited in geographic scope.

We framed the study from the Hutchinsonian perspective because doing so allowed us to: (1) measure how broad the niche dimensions of each species were; (2) compare the importance of different variables structuring the niche dimensions of the three species; and (3) evaluate whether potential responses of the species to shifting environmental conditions would likely be similar or more individualistic. We used Ecological Niche Factor Analysis (ENFA; Hirzel et al., 2002) to generate a model of niche space for each of these three species. We used these models to assess which ecogeographical variables were the primary drivers of current distributions and identify potential vulnerabilities from predicted environmental changes.

## 2 MATERIALS AND METHODS

### 2.1 Data collection

#### 2.1.1 Study area

**FIGURE 1:**
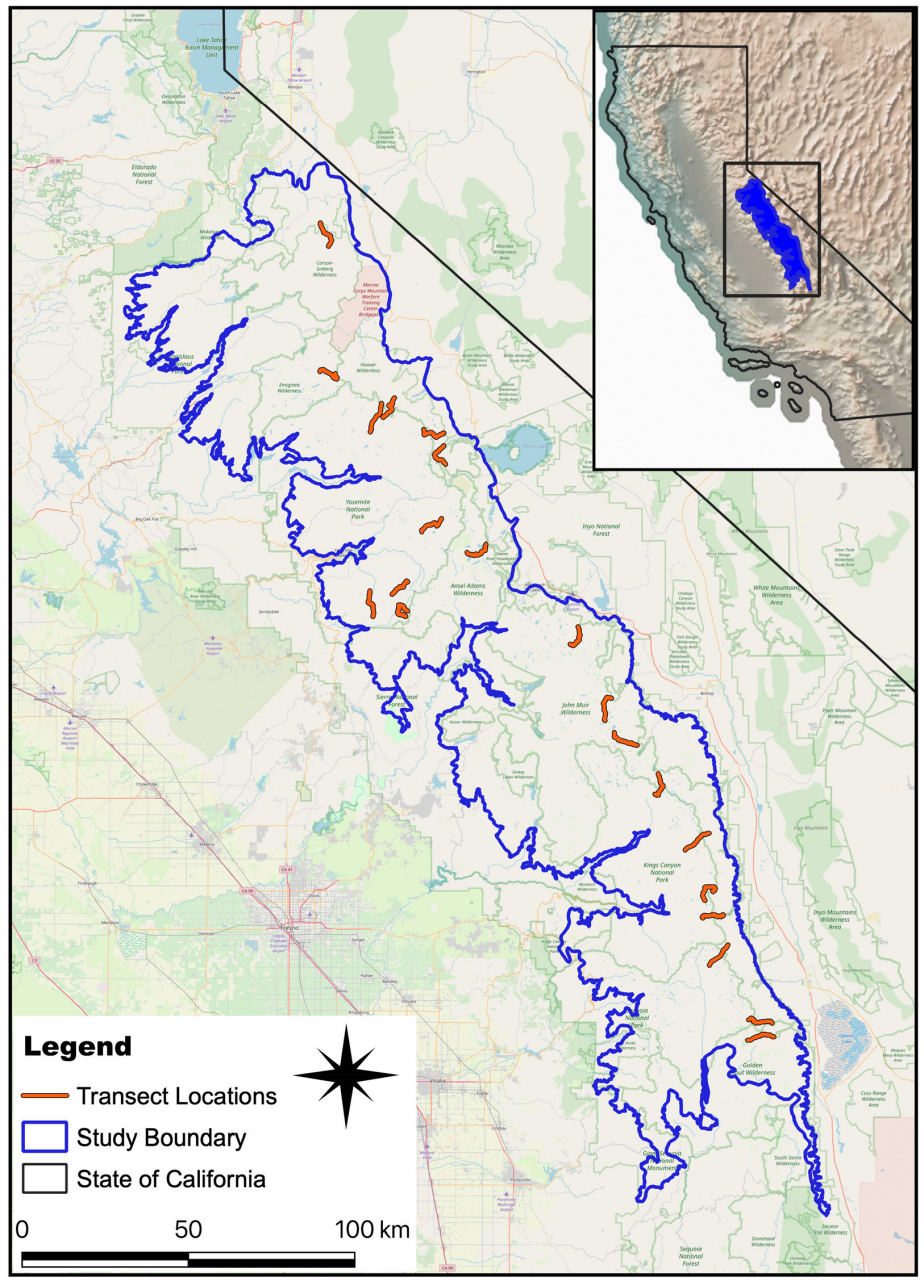
Study area boundary and transect locations. Inset depicts the study area location within California.

Our study was conducted in the alpine and upper subalpine zones of the central and southern Sierra Nevada (Sierra Nevada from hereon) and encompassed nearly all the alpine regions of the range. The study area spanned an elevation of 2500–3700 m along a north–south gradient of 320 km, from Alpine County in the north to the southern end of Tulare and Inyo counties in the south (Figure 1). The transition from subalpine to alpine decreases in elevation with increasing latitude (Fites‐Kaufman et al., 2007). The subalpine zone (2900–3660 m, with some regional variation) has a lower overall proportion of forested vegetation (25%) than the montane zone (70%–100%) below it, and comprises a mosaic of relatively sparse subalpine forests and woodlands, meadows, rock outcrops, and scrub (Fites‐Kaufman et al., 2007). Most of the annual precipitation occurs as snow (Fites‐Kaufman et al., 2007), and 95% of the precipitation falls between October and May (Storer et al., 2004). There can be periods during the summer when monsoon systems result in substantial precipitation from thunderstorms; however, near‐drought conditions often occur during the short summer growing season (Alden & Heath, 1998).

The upper subalpine and alpine zones are characterized by rocky slopes, low‐growing grasses and forbs, and patches of low‐statured conifers and shrubs (Archibold, 1995; Smith, 2000). Meadows are found within both zones and are defined by hydrology (shallow water table during the summer, generally <1 m from the surface), fine‐textured surficial soils, the dominance of herbaceous vegetation (woody plants may be present, but not dominant), and the presence of plants that require surface water, shallow groundwater, or both (Weixelman et al., 2011). In the upper subalpine zone of the Sierra Nevada, meadows are typically surrounded by conifers, whereas in the alpine zone conifer patches are embedded within a matrix of barren areas, rocky slopes, and meadows.

#### 2.1.2 Focal species

*Urocitellus beldingi* is a small‐bodied species (233 g; Smith et al., 2003) that occurs in the upper elevations (~1300–3500 m) in the Sierra Nevada (Grinnell & Dixon, 1918; Johnson, 2008; Morelli et al., 2012). *M. flaviventer* is a large‐bodied species (3400 g; Smith et al., 2003) that generally is found above 2200 m throughout much of the Sierra Nevada (Grinnell & Storer, 1924; Moritz et al., 2008). The *U. beldingi* and *M. flaviventer* reach the southern and western limits of their range in the Sierra Nevada (Frase & Hoffmann, 1980; Jenkins & Eshelman, 1984). *C. lateralis*, also a small‐bodied species (263 g; Smith et al., 2003), occurs at an elevational range of ~1370–3500 m (Grinnell & Dixon, 1918; Rowe et al., 2015). Although all three species are sympatric in much of our study area, these species have different geographic distributions, and available information indicates somewhat different habitat associations, suggesting they differ in factors determining their overall distributions. In particular, *M. flaviventer* and *U. beldingi* are thought to be meadow‐dependent (Jenkins & Eshelman, 1984; Svendsen, 1976), and *C. lateralis* is thought to be a meadow‐facultative species that also occurs in other habitats (Bartels & Thompson, 1993). Therefore, loss of meadows could be detrimental to all three of these species but particularly *M. flaviventer* and *U. beldingi*.

#### 2.1.3 Field sampling

We conducted our study over a large geographic area to encompass a broad range of the factors influencing the niches of these species. We conducted visual and auditory encounter surveys along 21 10‐km walking transects on established trails, randomly chosen from a pool of 68 existing trails, and separated by a minimum of 5 km (Figure 1). Most transects traversed the crest of the Sierra Nevada in a largely east–west orientation and avoided highly traveled routes such as the Pacific Crest Trail. Observers surveyed 12–21 transects three to four times each summer (mid‐June through late August) for 4 years (2009–2012), with repeat surveys conducted within one to 10 days of each other (median interval = 4 days). Surveys generally were conducted during the first 4 h after sunrise and the last 4 h before sunset but throughout the day when conditions favored continued activity by focal species (e.g., cloudy days). Each survey consisted of a single observer, walking the length of each transect at a steady pace. When a species was detected, the GPS location of the observer and the bearing and distance (measured with a laser rangefinder) to the species observation were recorded, allowing later calculation of the coordinate location of the species.

#### 2.1.4 Ecogeographical variables

We derived spatial data representing ecogeographical variables relevant to species distributions (e.g., climate, landcover class, topography, and vegetation production) from several sources (Table 1). Landcover data were obtained from the Classification and Assessment with Landsat of Visible Ecological Groupings (CALVEG) database (United States Department of Agriculture, 2012; 2.5 acres, ~1 ha minimum mapping unit). Climate data are monthly and were downloaded from the ClimSurf database (1950–2005; 860‐m resolution), including mean minimum (*T*_min_) and mean maximum (*T*_max_) monthly temperature, as well as total monthly precipitation (Precip; additional information about variables in Table 1). We used an updated version of the originally published ClimSurf climate model (Alvarez et al., 2014), which initially covered 1950–2000, and the updated version spanned the years 1950–2005 (personal communication O. Alvarez to R. Klinger). The number of days per year where snow comprised <15% cover (snow‐free days) was calculated from a fractional snow cover raster (2000–2010, 250‐m resolution; O. Alvarez and Q. Guo, University of California, Merced, unpublished data). Three topography variables were derived from a US Geological Survey (USGS) digital elevation model (DEM; 30‐m resolution): elevation, slope (degrees), and an index of topographic complexity (Terrain Ruggedness Index; TRI, United States Geological Survey, 2012b). Landsat data on the Normalized Difference Vegetation Index (Pettorelli, 2013) were used as an index of vegetation productivity (United States Geological Survey, 2012a; 1989–2012; 30‐m resolution).

**TABLE 1:** Description of the ecogeographical variables used in the Ecological Niche Factor Analysis, at a resolution of 30 m.

Variable name	Description
Elevation	Height above sea level (m)
Slope	Steepness of terrain
TRI	Terrain Ruggedness Index (Riley et al., 1999), the amount of elevation difference between adjacent cells of a digital elevation grid, a measure of topographic heterogeneity
Precip0609^a^	Precipitation during the summer season months, when precipitation primarily falls as rain, June–Sept
Precip1005^a^	Precipitation during the winter season months, Oct–May, when precipitation primarily falls as snow. October represents precipitation during early winter. January precipitation was strongly correlated with precipitation values in other winter and spring months (November through May), and so was considered a general representation of wetness for the mid to late winter season
Tmax07^a^	Mean (1950–2005) maximum temperature (°C) in July, representing the warmest month in the Sierra Nevada
Tmin01^a^	Mean (1950–2005) minimum temperature (°C) in January, representing the coldest month in the Sierra Nevada
Snow‐free days	Total days with <15% snow cover on the ground, averaged from 2000 to 2010
NDVI	Mean maximum of the Normalized Difference Vegetation Index (NDVI), a measure of vegetation conditions and forage productivity. 1989–2015
NDVICV	Coefficient of variation of the Mean Max NDVI, a measure of how variable vegetation conditions were 1989–2015
Meadow	CALVEG “herbaceous,” dominated by grasses and forbs
Rock	CALVEG “barren,” limited vegetation
Conifer	CALVEG, dominated by tree cover of conifers
Shrub	CALVEG Aspen, mixed shrub/tree, and shrub classes represented <5% of total land cover across the points, hence we pooled them together into a single class termed “shrub” because shrubs were the most common feature of the three classes

^a^ Climate data were derived from a downscaled climate layer (860 m^2^ resolution) developed at UC Merced from records spanning 1950–2005 (Alvarez et al., 2014).

Spatial data manipulation and analysis were conducted with the raster package (Hijmans & van Etten, 2012) in R (R Development Core Team, 2019). CALVEG landcover data (field Covertype) were converted to raster format for six classes: Conifer (conifer stands), Hardwood (predominantly groves of aspen, *Populus tremuloides*), Herbaceous (meadows), Mixed (interspersion of conifer and meadow), Shrub (low‐statured woody shrubs and willows, *Salix* spp.), and Rock (talus, rock fields). The Hardwood and Mixed classes comprised <1% of the study area, therefore we pooled them with Shrub and then calculated the proportion of each class (*N* = 4) per hectare. The landcover and climate rasters were resampled to a 30‐m resolution to match the resolution of the DEM. All spatial data layers of these environmental variables were combined into a raster stack (Hijmans & van Etten, 2012), and the values of the variables (Table 1) were extracted at each animal detection point and 50,000 random points. The available environmental conditions were represented through 50,000 random points (available habitat) within a 500 m belt on either side of each transect. The transects were limited to above 2500 m, so all random points below 2500 m were removed.

### 2.2 Data analysis

Ecological Niche Factor Analysis is a multivariate approach that computes environmental suitability functions for species based on ecogeographical variables (e.g., those representing topography, climate, and landcover; Hirzel et al., 2002). Ecogeographical variables often are correlated, and ENFA transforms these into uncorrelated factors of “marginality” and “specialization.”

Marginality is a measure of the magnitude of selection and is quantified as the amount of distance between the centroids of the modeled available niche space in the study region and that of the modeled used niche space occupied by the species. Higher marginality value indicates that a species' niche is increasingly different from the average conditions in the study region (Basille et al., 2008). Marginality is the first factor of the ENFA analysis. All the factors beyond marginality measure specialization. Specialization is a measure of niche breadth. Breadth of the niche is quantified by comparing the variance in a species' niche space to the variance in available environmental space (Hirzel et al., 2002).

The ecogeographical variables driving the measures of marginality and specialization indicate the environmental conditions related most strongly to each species. When the marginality coefficient for a variable is positive, this indicates that the species was found at a higher proportion of locations with those conditions than would have been expected based on the availability of those conditions on the landscape (Hirzel et al., 2002). The absolute value for the marginality indicates the magnitude of the effect of individual variables on selectivity for these species. The magnitude of the absolute value of the coefficient for specialization indicates the effect of that variable in restricting niche breadth (Hirzel et al., 2002); more restricted conditions for a variable than what is available are represented by smaller values.

All data manipulation and analysis were conducted in R (R Development Core Team, 2019), and the ENFA analysis was conducted using the “enfa” function in the “adehabitatHS” package (Calenge, 2006, 2011).

## 3 RESULTS

**FIGURE 2:**
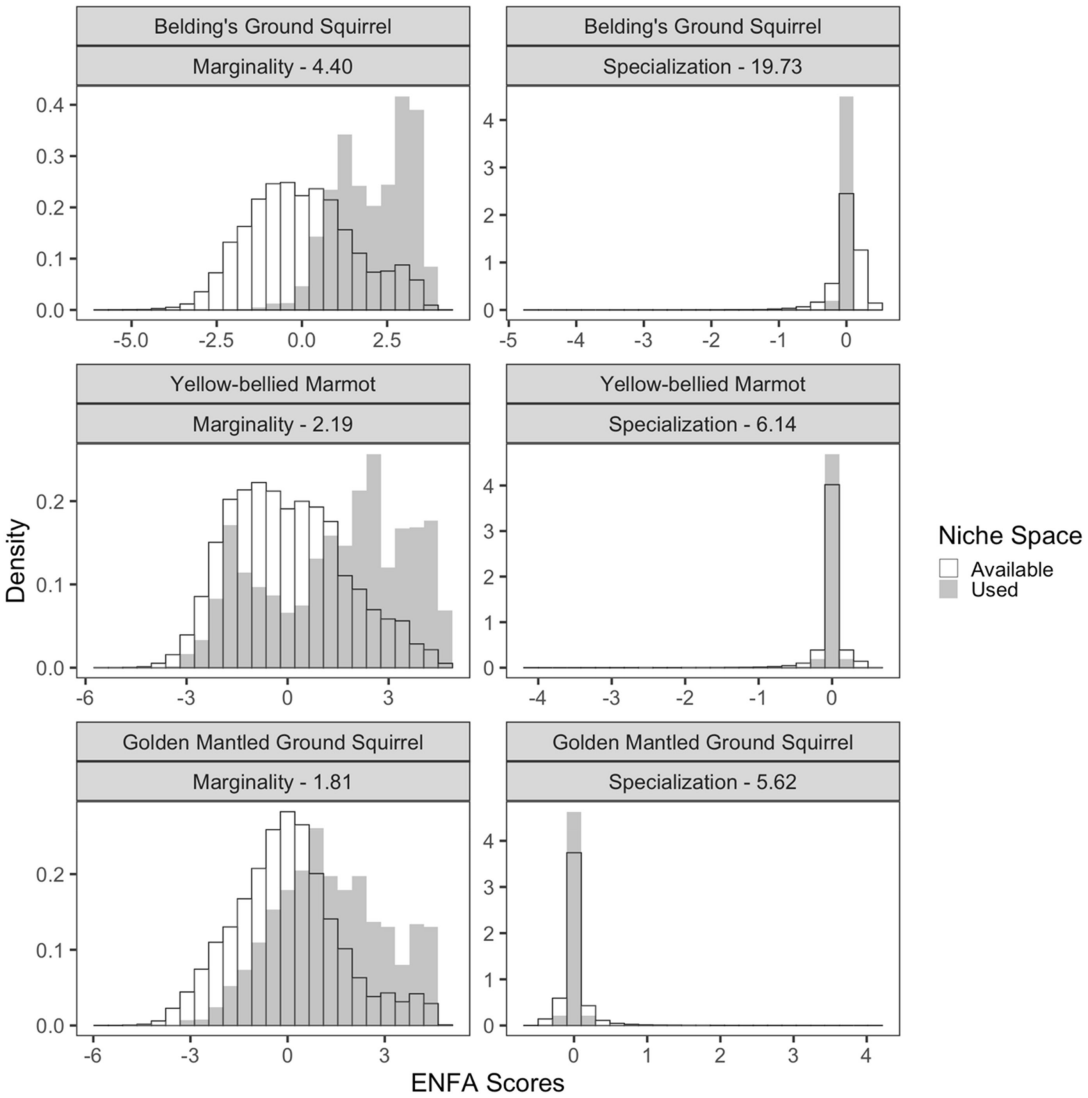
Comparison of the niche space of three species of mammals in the upper subalpine and alpine zones of the Sierra Nevada mountain range, USA. Marginality is a measure of the difference between the centroids of a species' niche space (shaded bars) and the available environmental space (open bars) as defined by 14 environmental variables (Table 3). Specialization is a measure of how restricted a niche is. Marginality is represented as the offset of shaded bars compared with open bars, and specialization by how narrow the shaded bars are compared with the open bars.

**FIGURE 3:**
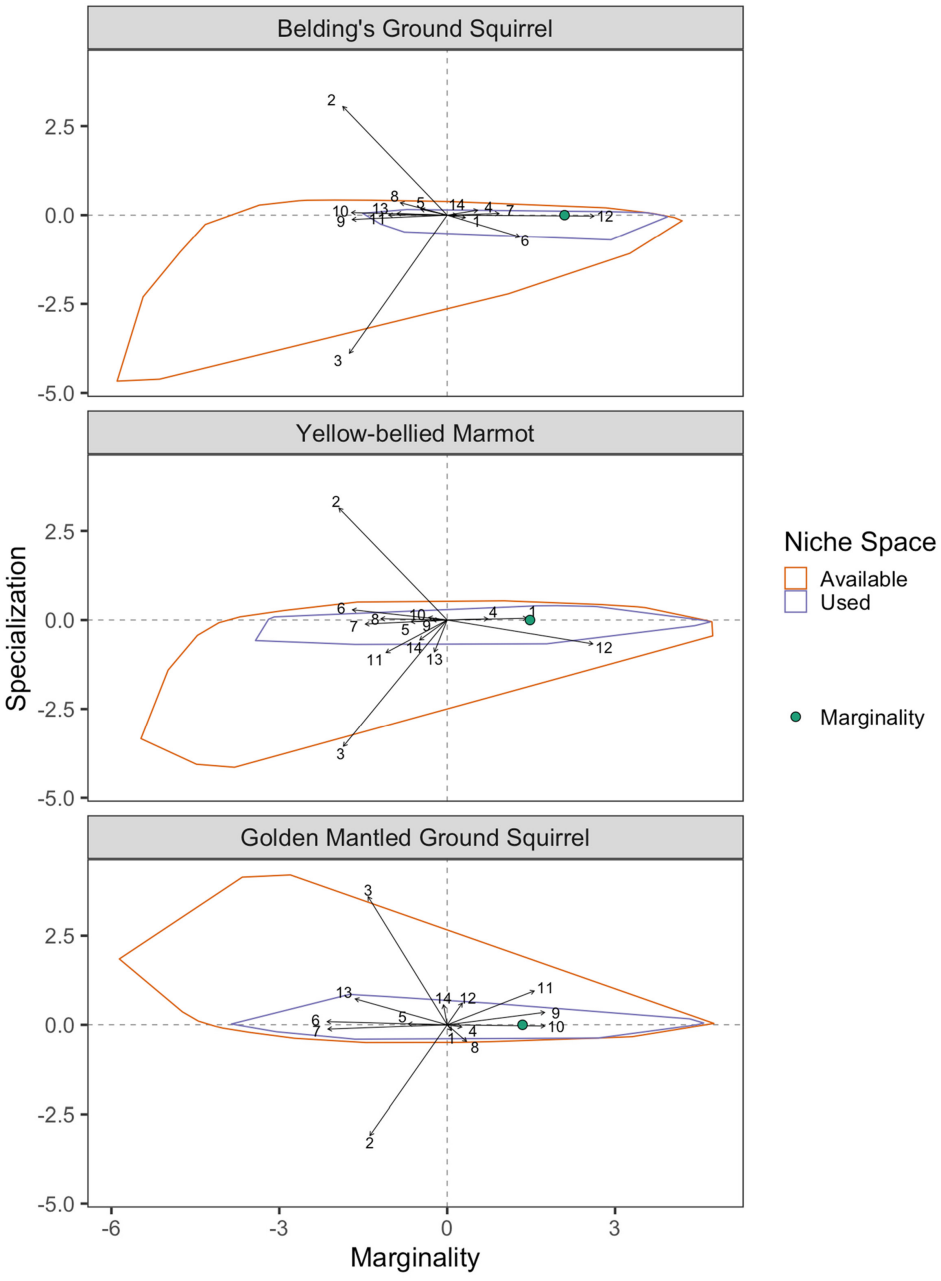
Biplot of the results of an Ecological Niche Factor Analysis (ENFA) for three species of mammals in the upper subalpine and alpine zones of the Sierra Nevada mountain range, USA. The orange polygon represents the available environmental space and the blue polygon represents the species' niche space. The center of the axes is the centroid of the available environmental space, the *x*‐axis is a measure of marginality, and the *y*‐axis is a measure of specialization. The shift of the centroid of the used niche space, shown by the teal dot, compared with the centroid of the available environmental space represents marginality. The vertical compression of the niche space compared with the available environmental space is a measure of how specialized the species is. Numbered variables are represented: 1. Elevation, 2. Slope, 3. TRI, 4. NDVICV, 5. NDVI, 6. Precip0609, 7. Precip1005, 8. Tmin01, 9. Tmax07, 10. Snow‐free days, 11. Conifer, 12. Meadow, 13. Rock, 14. Shrub.

We recorded a total of 5879 georeferenced observations (*M* = 1990): 2218 *U. beldingi*, 1703 *M. flaviventer*, and 2048 *C. lateralis* (Table 2). All three species showed selectivity in niche space, as reflected in the distribution of values for both marginality and specialization (Figures 2 and 3, Table 3). These measures were most pronounced for *U. beldingi* and least pronounced for *C. lateralis* (Figure 2), mainly because of the strong selection by *U. beldingi* for meadows (Figure 3). Niche space use for each of the species was driven by multiple variables in all three of the environmental categories (i.e., climate, landcover type, topography; Figure 3, Table 3).

**TABLE 2:** Sampling effort and detections by year for three species of mammals in the upper subalpine and alpine zones of the Sierra Nevada mountain range, USA.

Year	Transects	*U. beldingi*	*M. flaviventer*	*C. lateralis*
2009	12	346	145	239
2010	19	872	434	836
2011	21	596	456	464
2012	17	314	668	509

*Note*: Transects were 10‐km‐long × 1‐km‐wide (total area = 10 km^2^).

**TABLE 3:** Coefficients of the ecogeographical variables indicating the contribution of ecogeographical variables to marginality and specialization, by species.

Ecogeographical variable	Marginality			Specialization		
	*U. beldingi*	*M. flaviventer*	*C. lateralis*	*U. beldingi*	*M. flaviventer*	*C. lateralis*
Elevation	0.06	*0.28*	0.02	−0.01	0.01	−0.03
Slope	*−0.37*	*−0.39*	*−0.28*	**0.61**	**−0.63**	**−0.62**
TRI	*−0.35*	*−0.37*	*−0.28*	**−0.78**	**0.71**	**0.72**
NDVICV	0.11	0.15	0.05	0.03	0.01	−0.01
NDVI	−0.09	−0.13	−0.14	0.03	−0.01	−0.005
Precip0609	*0.26*	*−0.34*	*−0.43*	−0.12	0.06	0.02
Precip1005	0.19	*−0.29*	*−0.43*	0.01	0.02	−0.02
Tmin01	−0.17	*−0.24*	0.07	0.07	0.01	−0.09
Tmax07	−*0.34*	−0.05	*0.35*	−0.02	0.001	0.07
Snow‐free days	*−0.34*	−0.07	*0.35*	0.01	0.01	−0.01
Conifer	*−0.21*	*−0.22*	*0.31*	0.001	−0.18	0.19
Meadow	**0.52**	**0.52**	0.05	−0.01	−0.13	0.12
Rock	−0.18	−0.05	*−0.33*	0.01	−0.18	0.15
Shrub	0.03	−0.10	−0.01	0.001	−0.11	0.11

*Note*: See Table 1 for a description of the variables. To show the increasing importance of these different variables, values between |0.20–0.49| are shown in italics and values above |0.50| in bold. Positive values on the marginality indicate that the species is positively associated with higher values for that variable. This is not the case with specialization, and signs of the coefficient for specialization are irrelevant.

Marginality in *U. beldingi* was influenced primarily by the presence of meadows, although higher levels of both wet and dry season precipitation were also meaningful influences. Marginality was negatively correlated with the number of snow‐free days and higher maximum temperatures in summer, with a strong correlation between these two climate variables (Table 4). Marginality was negatively correlated with steep slopes and high levels of terrain ruggedness (Table 3). Similarly, the conifer and rock landcover types were negatively correlated with marginality for *U. beldingi*. Specialization for *U. beldingi* was influenced primarily by slope and terrain ruggedness (Figure 3), though there was some indication that dry season precipitation had a relatively weak influence as well (Table 3). Values of other coefficients in relation to specialization were low (Table 3).

**TABLE 4:** Correlation matrix for ecogeographical variables.

Elev	Slope	TRI	NDVI CV	NDVI	Precip 0609	Precip1005	Tmin 01	Tmax 07	Snow‐free days	Conifer	Meadow	Rock	Shrub
Elev	0.10	0.10	0.51	−0.53	−0.60	−0.65	−0.90	−0.49	−0.46	−0.29	0.15	0.30	−0.17
Slope		0.98	0.01	−0.06	−0.04	−0.06	−0.08	−0.01	0.01	−0.14	−0.29	0.39	0.10
TRI			0.01	−0.07	−0.03	−0.06	−0.08	−0.01	0.00	−0.15	−0.27	0.39	0.08
NDVICV				−0.86	−0.39	−0.50	−0.47	−0.09	−0.11	−0.19	0.17	0.10	−0.04
NDVI Mean Max					0.44	0.58	0.52	0.10	0.06	0.18	−0.15	−0.09	0.02
Precip0609						0.96	0.38	−0.29	−0.30	0.04	0.01	−0.07	0.03
Precip1005							0.47	−0.22	−0.27	0.08	−0.02	−0.07	0.00
Tmin01								0.73	0.60	0.35	−0.19	−0.29	0.11
Tmax07									0.83	0.32	−0.19	−0.25	0.11
Snow‐free days										0.33	−0.21	−0.27	0.15
Conifer											−0.34	−0.55	−0.29
Meadow												−0.27	−0.15
Rock													−0.22

*Note*: See Table 1 for a description of the variables.

Marginality in *M. flaviventer* was influenced primarily by a positive association with meadows and avoidance of areas with higher values for slope, terrain ruggedness, and conifers (Table 3 and Figure 3). Although our study was restricted to high‐elevation areas, higher elevations still had an important positive influence on *M. flaviventer* marginality. Overall levels of productivity (NDVI) had a negative correlation whereas variability in productivity (NDVICV) had a positive correlation with *M. flaviventer* marginality (Table 3). The coefficients of both NDVI variables were relatively small compared with those of most other variables. All the climate variables were negatively correlated with marginality in *M. flaviventer* (Table 3). In contrast with *U. beldingi*, marginality for *M. flaviventer* was negatively correlated with both wet and dry season precipitation levels. Slope and terrain ruggedness were the primary influences on specialization, though landcover types had a strong influence as well (Table 3; Figure 3). The other variables had little meaningful influence on specialization for *M. flaviventer* (Table 3).

Marginality in *C. lateralis* was strongly associated with climatic variables, especially precipitation (Table 3; Figure 3). They appeared to strongly select areas with lower levels of precipitation (wet and dry season), higher summer temperatures, and more snow‐free days (Table 3). Landcover variables also had strong influences on their marginality (Table 3), with strong evidence of selection for areas with conifers and few rocks (Figure 3). Landcover types also influenced *C. lateralis* specialization, but the strongest influences by far were related to topography (Table 3). Slope and terrain ruggedness (which had an extremely high correlation; Table 4) had strong effects on *C. lateralis* niche breadth (Figure 3). The influence of climate variables on *C. lateralis* specialization was negligible (Table 3).

## 4 DISCUSSION

Our study represents the first quantification and comparison of niche space for the three dominant sciurid species inhabiting one of the major montane ecosystems in the world. The analysis we used, Ecological Niche Factor Analysis (ENFA), quantified the niche and generated indices of “marginality” (magnitude of selection) and “specialization” (narrowness of niche space/niche breadth). Across numerous animal species, niche breadth is positively correlated with geographic range size (Brown, 1984; Slatyer et al., 2013). Our findings were consistent with this pattern; Belding's ground squirrels have the smallest range and most restricted niche of the three species (Jenkins & Eshelman, 1984), whereas the golden‐mantled ground squirrels have the largest range and broadest niche (Bartels & Thompson, 1993). Critically, our results were not confounded by sampling effects, where widespread species receive greater sampling effort (Slatyer et al., 2013). Our sampling effort was consistent among species, there was a comparable number of detections of each species, our study encompassed almost the entire extent of the alpine zone in the Sierra Nevada, and it occurred in an area of sympatry among the three species.

Environmental influences align closely with the concept of the Hutchinsonian niche, and ENFA allowed us to quantify this relative to features the three species selected (marginality) and environmental conditions that put strong constraints on their niche space (specialization). Two strong and consistent patterns were: (1) niche space was structured by a combination of topographic, landcover, and climate variables; and (2) as described in more detail below, the niche space was different for each of these three sympatric species, with some shared characteristics. These types of differences in niche space likely result in the variable response to climate change observed across taxa groups in montane regions (McCain & Garfinkel, 2021; Rowe et al., 2015; Tingley et al., 2009). The three species differed in the magnitude of selection along gradients that reflected their habitat use (with golden‐mantled ground squirrels being distinctly different than the other two), and indirectly their diet, but they were similar in niche breadth along topographical gradients. Topographical variables were the most important drivers of niche breadth for all three species.

Other factors besides environmental conditions can limit species distributions. The Sierra Nevada comprises the southern and western limits of the ranges of Belding's ground squirrels and yellow‐bellied marmots. One interpretation is that they are unable to tolerate environmental conditions beyond the Sierra Nevada. However, considerable care needs to be taken when assessing geographic distributions based just on a species realized niche (Soberón & Nakamura, 2009). Environmental conditions can be confounded with barriers to dispersal, and there are indications both Belding's ground squirrels and yellow‐bellied marmots have not dispersed to areas with apparently suitable meadow habitats in the Sierra Nevada. Belding's ground squirrels do not occur south of approximately 37.09°N latitude, even though there are many meadows well south of that. Yellow‐bellied marmots range further south than Belding's ground squirrels (approximately 36.18°N), but the limit of their range is also north of additional meadow habitat. Unsuitable environmental conditions and dispersal limitation are not necessarily independent, and we strongly suspect it is their interplay limiting the southwesterly distributions of both species. Our analysis shows that more precipitation was negatively associated with golden‐mantled ground squirrels and yellow‐bellied marmots but positively associated with Belding's ground squirrels, possibly showing a key distinction in niche space and possibly explaining different range limits at the southern portion of the range. Nevertheless, the reasonable possibility of dispersal limitation suggests that their niche dimensions might be broader than those represented by the ENFAs.

Conventional perspectives on the ecological niche have tended to investigate the conditions shaping species distributions separately from the functional role they play in ecosystems (Peterson et al., 2011). However, recent syntheses have combined habitat conditions, resources, and mechanisms of coexistence into a unified framework that explicitly recognizes how the environment shapes niche space, how species influence their environment, and how species interactions can influence their respective distributions (Letten et al., 2017). It was notable that the importance of particular ecogeographical variables on their niche axes differed among the three species we studied. More than just an outcome of individual variables though, differences in niche space among the species become clear when the magnitude of selection and niche breadth (measured as variable loadings on their marginality and specialization axes) are evaluated as an integrated whole among the species. This integration of their niche axes with the synthesis of Letten et al. (2017) provides a means of merging Hutchinsonian, Eltonian, and Grinnellian perspectives of the niche for interpreting: (1) Environmental influences on niche space under current conditions; and (2) Environmental influences on niche space under future climatic conditions.

### 4.1 Environmental influences on niche space under current conditions

#### 4.1.1 Magnitude of selection

Belding's ground squirrels and yellow‐bellied marmots are considered to occur predominantly in meadows and arid grasslands and feed mostly on grasses and forbs (Carey, 1985; Jenkins & Eshelman, 1984; Stallman & Holmes, 2002). Accordingly, in the Sierra Nevada, we found a strong selection for meadows by both species. However, the magnitude of selection differed between species along a precipitation gradient, Belding's ground squirrels were selective of moister conditions, whereas yellow‐bellied marmots were more selective of drier conditions. In contrast with the Belding's ground squirrels and yellow‐bellied marmots, golden‐mantled ground squirrels occur most frequently in conifer stands and consume seeds, as well as herbaceous plants (McKeever, 1964; Shick et al., 2006; Tevis, 1952; Trombulak, 1987). Golden‐mantled ground squirrels use margins of meadows, particularly those adjacent to conifer stands, as well as the edges of rocky slopes (Grinnell & Dixon, 1918; Hatt, 1927; Reichel, 1986). Consistent with those patterns, we found that in the Sierra Nevada, they were selective of conifer patches in relatively drier conditions and did not show strong use of meadows.

Although climate variables were important in some cases, the climate was not consistently the most important factor shaping the niche space of the three species. It is conceivable that this lower importance of climate could be due to the lower resolution of climate data when compared to our other variables. However, our study was limited to higher elevation regions with only modest climate variability at the macro‐scale we are evaluating, especially when compared to the variation in topography and landcover. It is more likely that the lower importance of climate variables was due to the entire study area being within or near the climate niche of these species. Nevertheless, even with this region with modest climate variability, the climate did influence their magnitude of selection. Climate can have direct (MacArthur & Wang, 1974; e.g., physiological stress; Beever et al., 2010) and indirect effects (Mantyka‐pringle et al., 2012; habitat transformation, novel biotic interactions, altered food resources; Blois et al., 2013; Wang et al., 2020) on species, and changing climate has influenced distributions in the Sierra Nevada (Rowe et al., 2015; Tingley et al., 2009). The ENFA indicated the likelihood that both effects are occurring in the Sierra Nevada, especially for Belding's ground squirrels and yellow‐bellied marmots. Belding's ground squirrels showed selection for more persistent snow cover, which points toward direct effects related to insulation from snow during hibernation (Johnston et al., 2021) or the importance of melting snowpack for providing moisture for meadow vegetation. But there was also an interplay between precipitation, meadows, and vegetation production for both Belding's ground squirrel and yellow‐bellied marmots, which is consistent with indirect effects on food quantity and quality (Andersson & Jonasson, 1986; Wei et al., 2019).

#### 4.1.2 Niche breadth

The consistently most important determinant of niche breadth for all three species was topographic complexity. The effect is likely due to the sites where they occurred being more open than those in the surrounding landscape, related to the conditions that support meadows, which are a food source for these species and depending on landcover can also offer good visibility.

In this high‐elevation region, climate variables were not meaningful determinants of the niche breadth. Marmots are stressed by high heat loads (Armitage, 2013), so it is surprising that their niche space is not compressed by temperature; however, it is not unexpected given our study area is limited to high‐elevation regions with only modest climate variability. Moreover, either through morphological (body size/shape, thick coats; Armitage, 2013; McCain & King, 2014), physiological (fat stores; Humphries et al., 2003), or behavioral (hibernation, activity times; Liow et al., 2009; McCain & King, 2014) traits, the species have the capacity to adjust, at least to a certain degree, to changing climatic conditions.

### 4.2 Environmental influences on niches under future climatic conditions

The emphasis put on the climatic component of a species' niche makes a tacit assumption that there is strong niche conservatism limiting the degree to which they can adapt to altered temperature and precipitation regimes (Pearman et al., 2008; Pyron et al., 2015; Saupe et al., 2019). However, there is evidence that genetically‐based constraints on niche space can be plastic, and there is support for species displaying local variation in adaptation to climatic change (Marcer et al., 2016; Pearman et al., 2010), including mammals that have often been considered vulnerable to changes in temperature and precipitation (Smith et al., 2019). Range contractions have not been observed for yellow‐bellied marmots over the past century, though upslope retractions of 244 m have been reported for golden‐mantled ground squirrels in the central Sierra Nevada over that period of time (Moritz et al., 2008; Rowe et al., 2015). Substantial range contractions have been forecast for Belding's ground squirrels (Morelli et al., 2012), and empirical patterns show they have undergone both downslope contractions from the upper elevational range (northern California) and upslope contractions from the lower elevational range (southern and central Sierra Nevada) over the past century (Moritz et al., 2008; Rowe et al., 2015). Interpretation of these patterns of expansion, retraction, or no change has been framed primarily from a climatic perspective, and in some instances with data that only represents a portion of their range. Forecasting changes in the distribution of a species should be done with data representative of the extent of conditions they encounter, not just a segment of it, and it should include more than just climate variables (Smith et al., 2019). The Sierra Nevada comprises only a portion of the ranges of the species we studied, so it is critical that evaluating the potential for changes in their distribution take into consideration their overall range, evolutionary history, dispersal, and flexibility in habitat selection behavior.

All three species range across portions of western North America that span relatively broad longitudinal and elevation ranges. Even though the Sierra Nevada does represent the edge of their geographic range, because these have encountered substantial spatiotemporal variation in climate in their evolutionary history, they might have the capacity to persist in the Sierra Nevada in the face of the changing climate. For example, most extant marmot species initially evolved at low elevations in the periglacial zone during the Pleistocene (Polly et al., 2015). The fossil record shows that marmots have tracked their climate niche by adjusting elevation over time (Davis, 2005; Polly, 2003; Tomé & Chaix, 2003), and they will persist in refugia with suitable climate conditions (Polly, 2003). Populations do occur at low elevations in the Great Basin (<1600 m), and across much of their range, they have persisted in areas and during times with warmer and drier conditions than in the Sierra Nevada (Floyd, 2004). There are indications yellow‐bellied marmots have disappeared from some small mountain ranges in the Great Basin, but most evidence points toward this being a natural extinction‐recolonization dynamic more so than climate‐driven (Floyd, 2004; Floyd et al., 2005). So, although mechanistic models do show a very high risk from climate change for this species (McCain, 2019), when that is placed in a broader context of their extensive range and evolutionary history there is an indication that they may have the capacity to persist in some regions even with increased temperatures.

In this study, we are intentionally evaluating niche determinants over broad temporal and spatial scales. The primary drivers of the niche might change (nonstationarity of the niche) if data were collected with conditions not included in our study, including no‐analog conditions, such as in a different region or during a different period of time (Bueno de Mesquita et al., 2021; García‐López & Allué, 2013). Measured determinants of distribution, abundance, and the niche space can vary over space, time, and scale (Beever et al., 2013; Jeffress et al., 2013; Johnston et al., 2019; Rossi, 2020). Although outside of the scope of this paper, this variation is also important for accurately predicting spatial response to changed conditions in topographically heterogeneous regions like the Sierra Nevada, where leading and trailing edges can occur within the overall range boundaries (Oldfather et al., 2020). Niche shifts can also occur through behavioral mechanisms, such as changes in habitat selection in response to environmental change. The results of our ENFAs reflect recent and current conditions, but we expect aspects of them, particularly the magnitude of selection (along the marginality axes), will change because of dynamic habitat selection as the three species respond to changing conditions. The magnitude of selection for niche space is closely aligned with habitat selection, so dynamic selection behavior would lead to variable loadings on the axes of marginality with different magnitudes than we documented in this study. Besides shifts in selection over time, the heterogeneous distribution of habitat conditions across a landscape can result in spatial variation, or functional response, in habitat selection (Godvik et al., 2009; Rossi, 2020). Therefore, it is important for future papers to consider variation in niche determinants, such as habitat selection variation over scale, space, and time.

## 5 CONCLUSIONS

High‐elevation species are thought to be among those most vulnerable to shifts in climate (Dirnböck et al., 2011; Parmesan, 2006), particularly these high‐elevation ground‐dwelling squirrels (McCain, 2019); therefore, an understanding of their niche is critical to better predict effects of changing conditions. Our findings indicate: (1) the consistent importance of topography and landcover on the niche space of the species, particularly in high‐elevation regions with comparatively limited macro‐climate variability. This underscores the importance of nonclimatic variables when predicting high‐elevation species distribution and abundance; and (2) the three species will demonstrate different responses to potentially climatically‐mediated changes in their environment. We used ENFA to understand dimensionality in the niches of the three dominant sciurid species in the high‐elevation zone of the Sierra Nevada, but it can also be used as a spatially explicit prediction tool (Rinnan & Lawler, 2019). Spatial predictions will often be a logical follow‐up to more explanatory‐based studies such as ours, but our results highlight why it is important to include other variables in addition to climate when spatial distribution models are used for predictive purposes.

## AUTHOR CONTRIBUTIONS

**Aviva J. Rossi:** Conceptualization (supporting); formal analysis (equal); project administration (supporting); validation (supporting); visualization (lead); writing – original draft (lead); writing – review and editing (lead). **Robert C. Klinger:** Conceptualization (lead); data curation (lead); formal analysis (lead); funding acquisition (lead); investigation (lead); methodology (lead); project administration (lead); supervision (equal); visualization (equal); writing – original draft (supporting); writing – review and editing (supporting). **Elise C. Hellwig:** Formal analysis (supporting); visualization (equal). **Dirk H. Van Vuren:** Conceptualization (supporting); formal analysis (supporting); funding acquisition (supporting); investigation (supporting); methodology (supporting); project administration (equal); resources (equal); supervision (lead); visualization (supporting); writing – original draft (supporting); writing – review and editing (supporting).

## Data Availability

Data from the project has been archived in accordance with USGS Survey Manual chapter SM 502.9—Fundamental Science Practices: Preservation Requirements for Digital Scientific Data. This guidance requires data to be preserved in accordance with the USGS records disposition requirements and the Federal Records Act 36 CFR 1220.14. Data can be found as “How will Mammals in the Alpine Zone of the Sierra Nevada Mountains Respond to Future Climate?” in the Science Base Catalog, within the National and Regional Climate Adaptation Science Centers, National Climate Adaptation Science Center, Fiscal Year 2009 Projects. Currently accessible at https://www.sciencebase.gov/catalog/item/4f833bd0e4b0e84f608680be.
